# Hip arthroscopy in patients with recurrent pain following Bernese periacetabular osteotomy for acetabular dysplasia: operative findings and clinical outcomes

**DOI:** 10.1093/jhps/hnv037

**Published:** 2015-06-13

**Authors:** Gregory L. Cvetanovich, Benton E. Heyworth, Kerri Murray, Yi-Meng Yen, Mininder S. Kocher, Michael B. Millis

**Affiliations:** 1. Rush University Medical Center, Department of Orthopaedic Surgery, 1611 W. Harrison Street, Suite 201, Chicago, IL 60612, USA and; 2. Children's Hospital Boston, Department of Orthopaedic Surgery, 300 Longwood Avenue, Fegan 2, Boston, MA 02115, USA

## Abstract

To report the operative findings and outcomes of hip arthroscopy for recurrent pain following periacetabular osteotomy (PAO) for acetabular dysplasia. A departmental database was used to identify patients who underwent hip arthroscopy following PAO between 2000 and 2009. Demographic data, arthroscopic findings, functional outcome scores and patient satisfaction were analysed. Of 556 PAO patients, 17 hips in 16 patients (3.1%) underwent post-PAO hip arthroscopy. Mean age at PAO was 23.8 years, and mean age at arthroscopy was 27.0 years. Common hip arthroscopy findings included labral tears (13 hips, 81.3%), significant (≥grade 2) chondral changes (12 hips, 75%), cam impingement (7 hips, 43.8%) and pincer impingement (6 hips, 37.5%). At mean follow-up 2.8 years after arthroscopy, additional procedures had been performed in six hips (37.5%), including total hip arthroplasty in one hip. Post-PAO arthroscopy questionnaire revealed 85.7% of patients with improved hip pain, 57.1% improved hip stiffness and 57.1% improved hip function. There was no significant difference in functional outcome measures. Common post-PAO hip arthroscopy findings include labral tears, chondral changes and femoroacetabular impingement. Many patients reported subjective hip improvement from post-PAO arthroscopy, but hip outcome scores were unchanged and one-third of patients had further surgery.

## INTRODUCTION

Acetabular dysplasia is an important cause of secondary osteoarthritis of the hip in the young adult, potentially necessitating hip arthroplasty at an early age [1–3]. For patients with acetabular dysplasia, the Bernese periacetabular osteotomy (PAO) is an effective surgical treatment which reorients the dysplastic acetabulum to stabilize the hip and to improve coverage of the femoral head [4–9]. Good long-term outcomes are achieved for the majority of patients, including a ‘preservation rate’ of hips ‘not’ requiring arthroplasty of 76% at a mean follow-up of 9-years [7] and 60% at a mean follow-up of 20-years [9]. Despite the benefits of PAO for the majority of patients, a subset of patients will experience recurrent pain after PAO [5]. Risk factors for poor outcomes following PAO include presence of moderate to severe pre-operative osteoarthritis, older age, presence of a labral tear, development of femoroacetabular impingement (FAI) after PAO, or presence of some features of FAI not addressed at the time of PAO, such as a cam lesion [8–12]. As a result, many surgeons perform PAO with concomitant hip arthrotomy in order to address labral pathology and femoral head-neck deformities that could lead to development of post-operative FAI [4, 7, 9, 13].

Hip arthroscopy has gained popularity in recent years due to favorable results and relatively low rates of complications [14, 15], with the most common indications including FAI, labral tears and loose bodies [16]. Notably, hip dysplasia is associated with high rates of labral pathology and chondral lesions [17–20]. Isolated arthroscopic treatment of intra-articular pathology in patients with acetabular dysplasia, however, has high rate of failure, likely because the central mechanical abnormality, instability, cannot be corrected by arthroscopic techniques [21, 22]. Because of this and concerns about additional morbidity from arthrotomy at the time of PAO, some authors have described acetabular osteotomy with simultaneous hip arthroscopy to address both the acetabular dysplasia and the associated hip intra-articular pathology [19, 20, 23].

For patients who have persistent or recurrent hip symptoms following PAO that are refractory to non-operative measures, hip arthroscopy is a potential treatment option, depending on the perceived etiology of the pain. However, there is limited data on the results of hip arthroscopy in this subpopulation of post-PAO dysplasia patients [7, 13].The purpose of this study was therefore to report the operative findings and post-operative functional outcomes of a series of patients at an academic referral center who had undergone prior PAO for acetabular dysplasia and subsequently underwent hip arthroscopy for hip pain. We hypothesized that patients with hip pain after PAO would have arthroscopic findings of treatable intra-articular pathology and improved hip outcome scores following arthroscopy.

## MATERIALS AND METHODS

### Study design and population

With institutional review board approval, a comprehensive hip procedural database at an academic referral center was used to retrospectively identify a series of patients who underwent hip arthroscopy for recurrent ipsilateral hip pain following previous PAO performed for acetabular dysplasia. PAO procedures were performed by a single surgeon between 2000 and 2009, with a previously described technique [7, 24]. Hip arthroscopy was performed by multiple surgeons; three surgeons at our institution performed a total of 13 of the post-PAO hip arthroscopies, and three surgeons from outside institutions performed a post-PAO hip arthroscopy, from which operative reports were obtained in all cases. The indications for hip arthroscopy after PAO included recurrent hip symptoms, such as pain, subjective complaints of instability without frank subluxation or dislocation, and/or snapping, in the post-PAO period that was refractory to conservative management. All patients reported improvement immediately following diagnostic intra-articular injections, but the improvement was transient in all cases, despite corticosteroid in the injection material. No such cases were performed for patients advanced osteoarthritic changes. We excluded patients for whom operative records for both PAO and hip arthroscopy were not available.

### Outcome measures

Data analysed included basic demographic information (gender, date of birth, age at time of PAO, age at time of post-PAO hip arthroscopy and laterality), preceding surgeries (number, type and documented surgical indications), radiographic findings, arthroscopic findings (cam lesion, pincer lesion, labral tear, chondral injury to the acetabulum or femoral head, synovitis, tear of ligamentum teres and psoas tendonitis), pre- and post-operative functional outcome measures, including University of California Los Angeles (UCLA) activity score [25], Modified Harris Hip Score (MHHS) [26] and Hip Disability and Orthoarthritis Outcome Score (HOOS) [27], which includes the Western Ontario (WOMAC) domains for pain, stiffness and function, and re-operations following arthroscopy. Magnetic resonance imaging (MRI) was obtained following PAO and prior to hip arthroscopy using 1.5 Tesla delayed gadolinium-enhanced magnetic resonance imaging (dGEMRIC) [28, 29]. Answers to an original questionnaire with questions specifically related to patients’ perceptions of their outcomes following the hip arthroscopy after PAO were also analysed. These included: (i) do you feel that your hip arthroscopy improved the hip pain that you were experiencing after the PAO?; (ii) do you feel that your hip arthroscopy improved the hip stiffness that you were experiencing after the PAO?; (iii) do you feel that your hip arthroscopy improved your hip function after the PAO? and (iv) are you satisfied today with the results of the surgeries that were performed on your hip?

### Statistical analysis

Demographic information for patients was summarized with means, standard deviations and ranges. Comparisons between outcome scores were via two-sample Student *t*-test with significance level *P* = 0.05 using SPSS Statistical Software (Chicago, IL).

## RESULTS

### Demographics

**Fig. 1.**
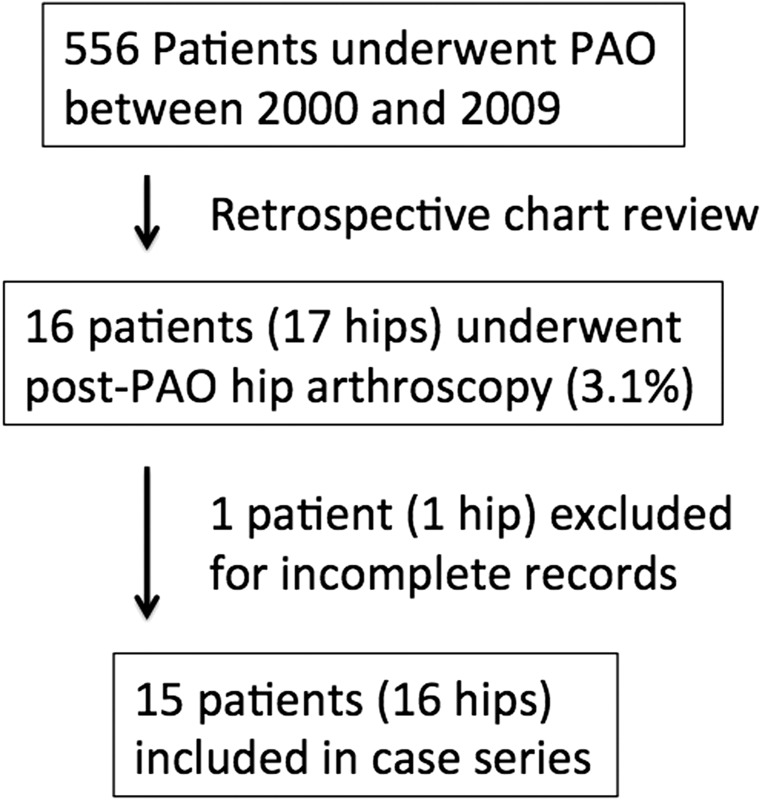
Flowchart demonstrating the selection of cases for the case series.

Of 556 patients undergoing PAO by a single surgeon at an academic referral center over a 10-year period with minimum 2-year follow-up following PAO, 16 patients (3.1%) and 17 hips underwent post-PAO arthroscopy. One patient with incomplete arthroscopy records from an outside hospital was excluded from the data analysis (Fig. 1). For the remaining 16 hips in 15 patients (13 females, 2 males; 9 right hip, 7 left hip, 1 bilateral), the mean age at time of PAO was 23.8 years (range 12.6–44.3 years), and the mean age at time of hip arthroscopy was 27.0 years (range 15.2–49.5 years), with a mean interval of 3.3 years (range 0.6–7.7 years) between PAO and hip arthroscopy. Two hips in two patients (12.5%) had undergone multiple surgeries for additional proximal femoral deformities prior to the PAO (Table I).

**Table I.** Demographic information for patients undergoing hip arthroscopy for recurrent symptoms after previous PAO

Demographics	Number
Male patients	2 (13.3%)
Female patients	13 (86.7%)
Left hip	7 (43.75%)
Right hip	9 (56.25%)
Bilateral hips	1 (6.25%)
Mean age at time of PAO in years (range)	23.8 (12.6–44.3)
Mean age at time of hip arthroscopy in years (range)	27.0 (15.2–49.5)
Time from PAO to hip arthroscopy in years (range)	3.3 (0.6–7.7)
Hips undergoing surgery prior to PAO for additional proximal femoral deformities	2 hips (12.5%)

### Operative findings during PAO

Of the 16 hips studied, nine hips (56.25%) underwent arthrotomy at the time of the PAO, which revealed labral tears in four hips, significant labral hypertrophy without labral tear in four hips and chondromalacia of the femoral head in four hips. Among these nine patients, four had multiple findings, four had only one significant finding and one had no findings. Six hips (37.5%) underwent additional procedures concurrent with the PAO: three hips underwent debridement of labral tears, one hip underwent greater trochanteric distal and lateral transfer for trochanteric overgrowth and three hips underwent intertrochanteric osteotomy (two for coxa valga and one for coxa vara) (Table II).

**Table II.** Findings at periacetabular osteotomy and concomitant procedures performed with PAO

Patient	Arthrotomy	Labral tear	Labral hypertrophy without tear	Femoral chondromalacia	Labral debridement	Greater trochanteric osteotomy	Intertrochanteric osteotomy
1						X	
2	X	X		X	X		X
3R							
3L							X
4							
5							
6	X	X		X			
7	X	X			X		
8	X		X				
9	X						
10	X	X		X	X		
11	X		X	X			
12	X		X				X
13							
14							
15	X		X				

### Radiographic and operative findings in post-PAO hip arthroscopy patients

All 15 patients (16 hips) who underwent hip arthroscopy after PAO had experienced recurrent hip symptoms, such as pain, subjective complaints of instability without frank subluxation or dislocation, and/or snapping, in the post-PAO period that was refractory to conservative management, including intra-articular steroid injections in all cases. Of note, all patients had undergone iliac crest screw removal at ∼6 months following PAO, according to the senior author’s protocol. Pre-arthroscopy radiographs and MRI were also performed in all cases. Pre-arthroscopy radiographs revealed anterior center edge angle 29.1 ± 6.4 (range 10–37), lateral center edge angle 26.4 ± 3.4 (range 20–33) and alpha angle 60.3 ± 10.0 (range 40–71). Tonnis grades were 0 in 2 hips, 1 in 11 hips and 2 in 3 hips. Anteroinferior iliac spine morphology based on false profile radiographs and axial MRI sequences was type II in 1 hip and type III in 1 hip, with the remaining 14 hips type I. Findings during hip arthroscopy included: labral tear (13 hips, 81.25%), ≥grade 2 chondral changes (12 hips, 75%), cam impingement (7 hips, 43.75%), pincer impingement (6 hips, 37.5%), synovitis (5 hips, 31.25%), torn ligamentum teres (1 hip, 6.25%) and psoas contracture and tendonitis (1 hip, 6.25%) (Table III). Of the 12 hips with significant chondral damage (≥grade 2), 10 lesions were identified on the femoral head (grade 2, *n* = 2; grade 3, *n* = 4; grade 4, *n* = 4) and 9 on the acetabular side (grade 2, *n* = 0; grade 3, *n* = 6; grade 4, *n* = 3) (Tables III and IV). Hip arthroscopy procedures performed were: labral tear debridement (12 hips, 75%), femoral head-neck osteochondroplasty (7 hips, 43.75%), acetabular osteoplasty (6 hips, 37.5%), synovectomy (4 hips, 25%), microfracture chondral defect (2 hips, 12.5%), labral repair (1 hip, 6.25%), debridement of ligamentum teres tear (1 hip, 6.25%) and psoas steroid injection to treat psoas tendinitis prior to closure (1 hip, 6.25%) (Table III).

**Table III.** Findings at hip arthroscopy after previous PAO and procedures performed during hip arthroscopy

Findings	Number of hips (%)
Cam impingement	7 (43.75%)
Pincer impingement	6 (37.5%)
Labral tear	13 (81.25%)
Synovitis	5 (31.25%)
Torn ligamentum teres	1 (6.25%)
Psoas contracture/tendonitis	1 (6.25%)
Chondral damage (≥ grade 2)	12 (75%)
Femoral head	10
Grade 2	2
Grade 3	4
Grade 4	4
Acetabulum	9
Grade 2	0
Grade 3	6
Grade 4	3
*Procedures performed*	*Number of hips (%)*

Labral tear debridement	12 (75%)
Labral repair	1 (6.25%)
Femoral head-neck osteochondroplasty	7 (43.75%)
Acetabular osteoplasty	6 (37.5%)
Debridement tear of ligamentum teres	1 (6.25%)
Microfracture chondral defect	2 (12.5%)
Synovectomy	4 hips, 25%)
Psoas steroid injection prior to closure	1 (6.25%)

**Table IV.** Arthroscopic cartilage grade and subsequent procedures after post-PAO arthroscopy

Patient	Femoral head ICRS grade	Acetabular rim ICRS grade	≥Grade 2 lesion (Y/N)	Procedure after arthroscopy
1	4	1	Y	Arthroscopy, labral debridement, femoral osteochondroplasty
2	4	0	Y	N/A
3R	2	3	Y	N/A
3L	0	1	N	Arthroscopy, labral debridement, femoral osteochondroplasty
4	3	3	Y	Resection of heterotopic ossification
5	1	4	Y	N/A
6	2	4	Y	Varus shortening intertrochanteric osteotomy for residual coxa valga with limb length discrepancy
7	0	0	N	Open femoral osteochondroplasty
8	1	0	N	N/A
9	0	0	N	N/A
10	4	4	Y	Total hip arthroplasty
11	4	3	Y	N/A
12	3	3	Y	N/A
13	3	3	Y	N/A
14	0	3	Y	N/A
15	3	1	Y	N/A

### Outcomes after post-PAO hip arthroscopy

At the time of final follow-up, additional procedures had been performed in six patients (37.5%) at a mean of 2.8 years following arthroscopy: open osteochondroplasty for residual FAI (one hip), secondary hip arthroscopy for labral debridement and osteochondroplasty (two hips), varus shortening intertrochanteric osteotomy for residual coxa valga with limb length discrepancy (one hip), resection of heterotopic ossification (one hip) and total hip arthroplasty (one hip). Otherwise, no complications of hip arthroscopy were reported.

Seven patients (46.67%) completed questionnaires specifically regarding satisfaction after hip arthroscopy following PAO at a mean post-arthroscopy follow-up of 5.7 years (range 1.1–9.0 years). Five patients (71.4%) had been satisfied for a period of time following PAO surgery. Six patients (85.7%) felt that the hip arthroscopy after PAO improved their hip pain. Four patients (57.1%) felt that the hip arthroscopy after PAO improved their hip stiffness. Four patients (57.1%) felt that the hip arthroscopy after PAO improved their hip function. Six patients (85.7%) were satisfied overall with their hip surgeries.

Table V shows the number of patients and hips who had available outcome scores as well as the mean functional outcome scores in the pre-PAO period, in the post-PAO/pre-arthroscopy period and in the post-arthroscopy periods. Final outcome scores were obtained a mean of 2.8 years (range 1.5–11.0 years) following arthroscopy. There were no significant differences between pre- and post-arthroscopy WOMAC, MHHS, HOOS and UCLA scores.

**Table V.** Functional outcome measures in the pre-PAO period, in the post-PAO/pre-arthroscopy period, and in the post-arthroscopy period

Pre-PAO	Number of hips (patients)	Mean Score ± SD (*P*-value)
UCLA	2 (2)	4 ± 0
MHHS	2 (2)	64.9 ± 7.78
HOOS	2 (2)	49.7 ± 29.6
WOMAC pain	16 (15)	7.4 ± 4.5
WOMAC stiffness	16 (15)	2.4 ± 2.1
WOMAC function	16 (15)	21.9 ± 14.4
**Post-PAO/Pre-arthroscopy**		
UCLA	6 (6)	6.3 ± 3.2 (0.37)
MHHS	6 (6)	59.2 ± 7.9 (0.41)
HOOS	5 (5)	64.4 ± 13.6 (0.37)
WOMAC pain	14 (13)	6.9 ± 3.8 (0.75)
WOMAC stiffness	15 (14)	2.1 ± 1.7 (0.67)
WOMAC function	15 (14)	15.4 ± 12.0 (0.18)
**Post-arthroscopy**		
UCLA	14 (13)	6.1 ± 1.9 (0.86)
MHHS	15 (14)	66.6 ± 12.0 (0.18)
HOOS	12 (12)	61.2 ± 19.0 (0.74)
WOMAC pain	15 (14)	7.4 ± 4.1 (0.74)
WOMAC stiffness	15 (14)	3.0 ± 1.4 (0.13)
WOMAC function	12 (12)	19.5 ± 15.0 (0.43)

WOMAC, Western Ontario domains for pain, stiffness and function.

## DISCUSSION

This study investigated the intra-operative findings and post-operative outcomes of a series of 16 hips in 15 patients at an academic referral center who had undergone prior PAO for acetabular dysplasia and subsequently underwent hip arthroscopy for recurrent hip pain refractory to conservative measures. Two previous studies discussed outcomes of smaller cohorts of similar patient sub-populations. In a retrospective study comparing patients undergoing PAO alone versus patients undergoing PAO with combined open femoral head-neck junction osteochondroplasty, Nassif *et al*. [13] reported that subsequent hip arthroscopy for suspected FAI or labral pathology was performed in 4 of 48 (8.3%) patients in their PAO group and in 1 of 40 (2.5%) patient in their PAO with osteochondroplasty group at a mean follow-up of 2.8 years. Of these five patients, all five had labral tears, of whom four underwent labral repair and one underwent labral debridement. In addition, two patients (both of whom had not had osteochondroplasty at the time of initial procedure) were felt to have cam lesions of the femoral head-neck junction and underwent arthroscopic osteochondroplasty to address the cam lesion. Their reported arthroscopy after PAO rate (5.7% combining their two groups) was similar to that in this study (3.1%). In addition, the findings of labral tears and cam lesions were similar between the two studies. Notably, however, in the current investigation pincer lesions and chondral changes in the femoral head and acetabulum were seen in a substantial number of patients but not identified in any patients by Nassif *et al*., which may be related to reporting bias or differences in sample size.

In a previous study focused on the long-term outcomes of PAOs performed from 1991 to 1998, Matheney *et al*. reported that 15 hips (11% of the total PAOs studied in that period) underwent hip arthroscopy for debridement of labral or chondral lesions at a mean of 6.8 years after PAO [7, 24]. Although outcome measures within this sub-group were not separately analysed, as in this study, the authors did report on operative findings of arthroscopy. In the previous report, 13 hips had at least partial thickness femoral cartilage lesions, of which four had full-thickness femoral cartilage lesions. Thirteen hips also had at least partial loss of acetabular cartilage, of which three had full-thickness acetabular cartilage loss. All 15 had labral tears that underwent debridement. One hip went on to total hip arthroplasty in their series. Overall, the operative findings of post-PAO arthroscopy in the cases reported by Matheney *et al.* are similar to those in this study. The 11% rate of arthroscopy after PAO is significantly higher than the 3.1% post-PAO hip arthroscopy rate (*P* < 0.05) in this study and may be most specifically attributable to the difference in duration of follow-up, though advances in PAO technique or improved patient selection between the two study periods may also be confounding factors. For instance, improved methods of imaging cartilage such as dGEMRIC may improve patient selection for PAO [28, 29]. While concomitant arthrotomy during PAO was performed in 56% of patients in the currently studied population, compared with 61% in that studied by Matheney *et al.,* improved methods for addressing concomitant intra-articular pathology at the time of PAO, could also be related to lower rates of subsequent hip arthroscopy.

Several reports of small series of patients who underwent hip arthroscopy following pelvic osteotomies other than PAO for acetabular dysplasia have also emerged. Ilizaliturri *et al*. [30] reported on a series of seven patients who underwent hip arthroscopy for mechanical hip symptoms following prior Chiari osteotomy. In all seven patients, massive labral tears and associated acetabular and femoral cartilage damage were seen, which were attributed to the medial displacement of the acetabulum resulting from the Chiari osteotomy. Klein *et al*. [31] described a case series of three patients who underwent Pemberton pelvic osteotomy for hip dysplasia, returning an average of 12 years after osteotomy with hip pain secondary to labral damage, based on arthroscopic assessment. Fujii *et al.* [19] performed transposition osteotomy of the acetabulum with concomitant hip arthroscopy in 23 hips in 22 patients, as well as second look arthroscopy in 13 hips in 12 patients. They found that about 85% of intra-articular findings at time of original osteotomy were unchanged at time of second-look arthroscopy a mean of 18.2 months later, with findings including cartilage lesions of the femoral head and acetabulum as well as labral pathology. It is difficult to compare the findings of these studies to this study because the associated intra-articular pathology of these populations are likely to be different and the osteotomies are technically different from the Bernese PAO.

A strength of the current series is the inclusion of multiple validated functional hip outcome scores following hip arthroscopy after prior PAO. Nassif *et al.* [13] reported that their patients’ symptoms improved, but did not assess objective functional outcome scores. Matheney *et al*. [7, 24] found that after post-PAO arthroscopy, the WOMAC pain score was >10 in four hips and an average of 1.7 in the other patients, with one patient going on to total hip arthroplasty. This is similar to our findings of a mean WOMAC pain score of 7.4 with one hip going on to total hip arthroscopy. However, Matheney *et al*. did not specifically compare pre-arthroscopy and post-arthroscopy outcome scores to investigate the specific effect of arthroscopy. Our data are consistent with Nassif *et al.*, in that the majority of patients reported subjective symptomatic improvement. However, we found that when more rigorous objective function outcome metrics for the hip were analysed, there were no significant improvements following hip arthroscopy. In addition, we found that roughly one-third of patients went on to have additional procedures after arthroscopy, much of which could be related to residual FAI as evidenced by high alpha angles on pre-arthroscopy radiographs, to degenerative changes of the hip based on pre-arthroscopy Tonnis grade of 2 in 3 hips, and to high rates of high-grade chondral damage identified at hip arthroscopy after PAO. However, it is possible that the lack of improvement in hip outcome scores could in fact reflect that hip arthroscopy prevented patients’ hip scores from worsening. To address this concept, a case control or randomized controlled trial would better address the question of whether hip arthroscopy was beneficial in patients with recurrent symptoms after prior PAO.

### Limitations

Limitations of this study include the retrospective case series design with a heterogeneous patient population and the confounding factor of inconsistency within the cohort of whether concurrent hip arthrotomy was performed at the time of PAO. For example, the seven hips that did not undergo arthrotomy may have contained untreated intra-articular pathology that led to the need for subsequent hip arthroscopy. However, the majority of the series included patients that did undergo arthrotomy, so it is unclear whether some of the reported hip pain stemmed from new lesions that developed following the time of arthrotomy. The lack of a matched control group of conservative treatment is a limitation, although reporting the outcomes of this rare population of patients who underwent prior PAO, had recurrent hip pain refractory to conservative management, and underwent hip arthroscopy limited us to a retrospective case series level IV design. An additional limitation is that several of the current series’ hip arthroscopies were performed by surgeons at outside institutions, resulting in variation in technique and the information available in operative reports. We also did not consider extra-articular impingement including iliopsoas impingement, subspine impingement and ischiofemoral impingement as an additional dynamic source of hip pain that has been increasingly recognized in recent years. Many patients had missing outcome score data particularly for the pre-operative timepoint, which limited our ability to analyse outcome scores of arthroscopy after PAO. Finally, only approximately half of patients returned the questionnaire regarding about satisfaction with the hip arthroscopy after PAO, which could result in non-respondent bias if those who returned the survey were more inclined to be satisfied or dissatisfied with their arthroscopy compared with those who didn’t return the survey.

## CONCLUSIONS

For patients with recurrent hip symptoms following PAO, common hip arthroscopy findings include labral tears, chondral changes and FAI. At a mean follow-up of ∼3 years, while many patients reported that post-PAO hip arthroscopy improved their hip pain, function and stiffness, no significant improvements were seen in hip outcome scores, and approximately one-third of patients went on to have additional procedures.

## CONFLICT OF INTEREST STATEMENT

None declared.
